# Breast Carcinomatous Tumoral Emboli Can Result From Encircling Lymphovasculogenesis Rather Than Lymphovascular Invasion

**DOI:** 10.18632/oncotarget.117

**Published:** 2010-06-10

**Authors:** Sepi Mahooti, Kyle Porter, Mary L. Alpaugh, Yin Ye, Yi Xiao, Susie Jones, Joseph D. Tellez, Sanford H. Barsky

**Affiliations:** ^1^Department of Pathology and Center for Biostatistics, The Ohio State University College of Medicine, Columbus, Ohio 43210; ^2^Center for Biostatistics, The Ohio State University College of Medicine, Columbus, Ohio 43210; ^3^Memorial Sloan-Kettering Cancer Center, New York, NY 10065; ^4^University of Nevada School of Medicine, Reno, NV 89557; ^5^Nevada Cancer Institute, Las Vegas, NV 89135

## Abstract

The canonical view of the origin of tumor lymphovascular emboli is that they usually originate from lymphovascular invasion as part of a multistep metastatic process. Recent experimental evidence has suggested that metastasis can occur earlier than previously thought and we found evidence that tumor emboli formation can result from the short-circuiting step of encircling lymphovasculogenesis. Experimentally, we used a xenograft of human inflammatory breast cancer (MARY-X), a model that exhibited florid tumor emboli, to generate tumoral spheroids *in vitro*. In observational studies, we chose human breast carcinoma cases where there appeared to be a possible transition of *in situ* carcinoma to lymphovascular emboli without intervening stromal invasion. These cases were studied by morphometry as well as IHC with tumor proliferation (Ki-67) and adhesion (E-cadherin) markers, myoepithelial (p63), as well as endothelial (podoplanin [D2-40], CD31, VEGFR-3, Prox-1) markers. Unlabelled spheroids coinjected with either GFP or RFP-human myoepithelial cells or murine embryonal fibroblasts (MEFs) gave rise to tumors which exhibited GFP/RFP immunoreactivity within the cells lining the emboli-containing lymphovascular channels. *In vitro* studies demonstrated that the tumoral spheroids induced endothelial differentiation of cocultured myoepithelial cells and MEFs, measured by real time PCR and immunofluorescence. In humans, the *in situ* clusters exhibited similar proliferation, E-cadherin immunoreactivity and size as the tumor emboli (p =.5), suggesting the possibility that the latter originated from the former. The *in situ* clusters exhibited a loss (50%-100%) of p63 myoepithelial immunoreactivity but not E-cadherin epithelial immunoreactivity. The tumor emboli were mainly present within lymphatic channels whose dual p63/CD31, p63/D2-40 and p63/VEGFR-3 and overall weak patterns of D2-40/CD31/VEGFR-3 immunoreactivities suggested that they represented immature and newly created vasculature derived from originally myoepithelial-lined ducts. Collectively both experimental as well as observational studies suggested the possibility that these breast cancer emboli resulted from encircling lymphovasculogenesis rather than conventional lymphovascular invasion.

## INTRODUCTION

The canonical view of human cancer progression is that *in situ* carcinoma progresses to invasive carcinoma which become lymphovascular emboli that metastasize. It is widely accepted that metastasis is a late event in cancer progression. Recent studies in transgenic mice have observed that metastasis can occur earlier in tumor progression than previously thought [1]. Possible explanations for “early metastasis” include passive dissemination of tumor cells by iatrogenic mechanisms, synchronous or metachronous transformation of stem or progenitor cells present in diverse locations and the ability of *in situ* or incipient cancers to bypass many of the traditionally rate-limiting steps of the metastatic process which include stromal invasion, angiogenesis, lymphangiogenesis, intravasation and extravasation [2-8].

Lymphovascular invasion (LVI), lymphangiogenesis and vasculogenesis are all important steps in tumor metastasis and these steps in themselves can occur through several different mechanisms [9-14]. One such mechanism is vasculogenic mimicry [15-21]. This type of mimicry is exhibited by several experimental models of tumor progression whereby tumor cells directly differentiate into vascular channels and bypass the reliance on conventional angiogenesis [17-19]. Another potential mechanism of bypassing conventional angiogenesis is the recruitment of local or bone marrow-derived stem cells to participate in vasculogenesis [20]. Along these lines, we searched for evidence to suggest that some of the steps of human cancer progression could be side-stepped so that human cancers would be positioned to metastasize earlier in their natural history. The evidence that we found and present in this study suggests that the step of tumor emboli formation results from the short-circuiting step of encircling lymphovasculogenesis rather from the classic step of lymphovascular invasion.

## RESULTS

### Experimental studies

#### Animal studies

**Figure 1.**
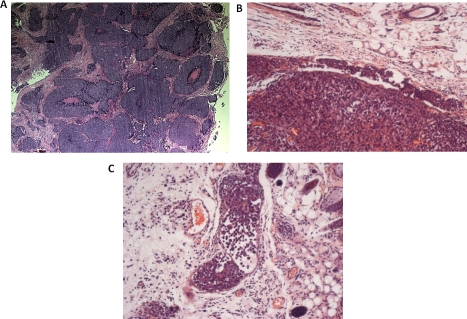
Histopathology of MARY-X (A) MARY-X exhibits a centrally prominent nodular growth pattern. (B) Peripheral to the main mass, a tumor embolus within an adjacent lymphovascular space is observed. (C) Florid LVI of overlying dermal lymphatics is observed.

**Figure 2.**
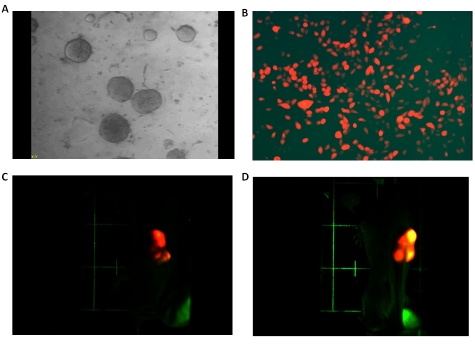
MARY-X spheroids with fluorescently labeled HMS-1/MEFs (A) MARY-X gives rise to spheroids *in vitro* which consist of very tight aggregates of tumor cells in suspension culture. (B) Retroviral transfection and G418 clonal selection gives rise to HMS-1 clones expressing strong RFP. (C) Coinjection of MARY-X spheroids with RFP-HMS-1 cells in GFP-transgenic nudes gives rise to tumors initially expressing red fluorescence. Identical results were observed when the spheroids were coinjected with RFP-MEFs. (D) Over time the red-fluorescing nodules (MARY-X spheroids + RFP-HMS-1) depicted previously expressed a hybrid yellow fluorescence.

**Figure 3.**
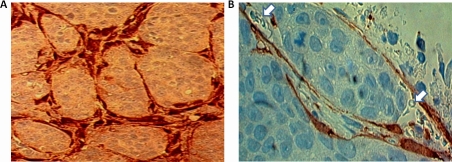
MARY-X coinjection immunocytochemical studies (A) Immunocytochemical studies of an extirpated tumor coinjected with GFP-labeled MEFs reveals tumoral nodules surrounded by encircling GFP immunoreactivity. Identical results were observed when the spheroids were coinjected with GFP-HMS-1. (B) Higher magnification of these tumoral nodules reveals their location within a space containing GFP immunoreactivity in its lining and luminal erythrocytes (arrows).

Our xenograft model of human inflammatory breast cancer (MARY-X), a model that exhibited a nodular growth pattern in its center (Figure 1A), LVI at its periphery (Figure 1B) with involvement of adjacent dermal lymphatics (Figure 1C) exhibited large numbers of spontaneous pulmonary metastases. The xenograft generated numerous tumoral spheroids *in vitro*. Unlabeled MARY-X tumoral spheroids (Figure 2A) coinjected with either GFP or RFP-labeled human myoepithelial cells (HMS-1) (Figure 2B) or MEFs into nude mice gave rise to tumors which exhibited GFP or RFP fluorescence. Green or red fluorescence was observed within the emerging tumor nodules based on the coinjected GFP or RFP-labeled HMS-1 or MEFs. Interestingly in the experiments involving the mixture of unlabeled spheroids with RFP-labeled HMS-1 or MEFs in GFP-transgenics, the tumor nodules initially exhibited a red fluorescence (Figure 2C) which over time gave rise to a hybrid yellow fluorescence (Figure 2D). On IHC examination of the extirpated tumor nodules, circumferential GFP or RFP immunoreactivity was observed surrounding the tumor cell clusters (Figure 3A). Unlabeled MARY-X tumoral spheroids coinjected with either GFP or RFP-labeled HDFs gave rise to tumors which did not fluoresce. On IHC examination of the extirpated tumor nodules, no GFP or RFP encircling immunoreactivity was observed.

These present studies are immunocytochemical studies and not fluorescence studies (Figure 3A; Figure 3B). Although GFP and RFP fluoresce green and red colors respectively when subjected to UV light, under white light they do not fluoresce. In analyzing the extirpated xenografts we used rabbit polyclonal antibodies to GFP and RFP and secondary goat anti-rabbit antibodies conjugated with DAB, giving a brown color at sites of GFP or RFP. The brown color demonstrated reflects the presence of GFP (Figure 3A; Figure 3B). The GFP immunoreactivity shows brown staining around the clusters of tumor cells, hence it shows encircling immunoreactivity (Figure 3A). In particular, the brown-colored cells appear to be lining a space which surrounds a clump of tumor cells (Figure 3B). When one looks carefully at this space, the presence of luminal erythrocytes (red blood cells) is noted suggesting that this space is indeed a lymphovascular space.

#### In vitro studies

**Figure 4.**
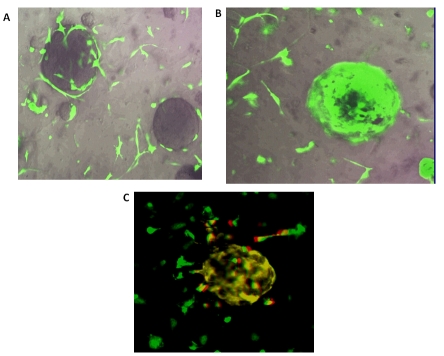
*In vitro* coculture fluorescent studies (A) Coculture experiment with Matrigel viewed with the Fluorescence Imaging Microscopy-Nikon Eclipse TE2000-U System revealed the GFPlabeled MEFs migrating toward the unlabeled spheroids of MARYX. (B) Over the next 72-96 hr, the GFP-MEFs completely encircled the spheroids. (C) Applying a rat anti-mouse CD31 antibody followed by a Texas red-conjugated rabbit anti-rat indicates hybrid yellow CD31 expression by the GFP-MEFs spheroid-encircling cells. Isolated MEFs themselves were largely negative for CD31 (mainly green). Fluorescent patterns were similar when GFPHMS-1 were cocultured with the spheroids and a rat anti-human CD31 was used.

Experiments with either GFP-labeled HMS-1 or GFP-labeled MEFs cocultured with unlabeled MARY-X spheroids in Matrigel revealed prominent chemotaxis and haplotaxis of the HMS-1 or MEFs toward the spheroids (Figure 4A) with near complete encircling of either of the former cells around the spheroids by 72-96 hrs (Figure 4B). Antibodies against human-specific or murine-specific CD31 revealed expression of this molecule on the HMS-1 or MEFs respectively after they had surrounded the spheroids (Figure 4C). The detection system utilized a Texas-red conjugated secondary antibody which produced hybrid yellow fluorescence in the GFP-labeled HMS-1 or MEFs (Figure 4C). Both the HMS-1 cells and the MEFs were essentially negative for CD31 immunoreactivity when cultured in isolation or when they were not encircling the MARY-X spheroids (Figure 4C).

The vast majority of individual cells outside of the spheroid showed only green fluorescence (Figure 4C). Although rare outside cells showed red and yellow fluorescence, clearly the cells that encircled the spheroid showed much more intense yellow fluorescence. Therefore this data clearly showed the acquisition of CD31 immunoreactivity in the cells that have encircled the spheroid. Although it is possible that this yellow color was the result of a dual population of cells, one with green fluorescence only and the other exhibiting red (CD31) immunofluorescence only, we feel that this alternative explanation is not likely because outside of the spheroid, the vast majority of cells exhibit green fluorescence and only rare numbers of cells exhibiting red and yellow immunofluorescence are observed. Therefore it is more likely that the GFP-labeled cells acquire CD31 immunoreactivity when they encircle the spheroid and change to a yellow color. A single population of cells then emit both green fluorescence and acquire this red immunofluorescence producing a hybrid yellow color.

**Figure 5.**
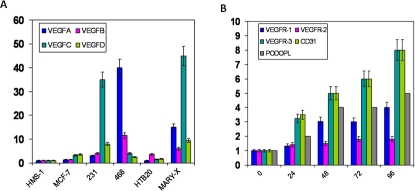
Real time PCR for endothelial growth factors and receptors (A) Real time PCR of relative mRNA levels of the VEGF family (VEGF-A, VEGF-B, VEGF-C, VEGF-D) for different breast carcinoma cell lines and the spheroids of MARY-X. Results depicted are a mean + standard deviation of 5 experiments. (B) Real time PCR of relative mRNA levels of the VEGFR family (VEGFR-1,VEGFR-2, VEGFR-3), CD31 and podoplanin over time (hr) for HMS-1 cells cocultured in media with MARY-X spheroids. Results depicted are a mean + standard deviation of 5 experiments.

MARY-X spheroids prior to coculture were harvested and compared to a series of common breast carcinoma cell lines with respect to mRNA transcript levels by real time PCR of the VEGF family of growth factors. The HMS-1 myoepithelial cell line was used as control. The ER negative lines (MDA-MB-468, MDA-MB-231) exhibited higher levels of VEGF-C and VEGF-A expression respectively than the ER positive lines (MCF-7, HTB20) (Figure 5A). MARY-X spheroids also exhibited very high levels of VEGF-C and reasonably high levels of VEGF-D (Figure 5A), compared to other line.

Experiments with HMS-1 cocultured with unlabeled MARY-X spheroids in regular culture media revealed an interesting pattern of induction of gene expression in the HMS-1 cells. Members of the VEGFR family (VEGFR-1, VEGFR-2, VEGFR-3), CD31 and podoplanin all showed increased transcript levels over time of coculture by real time PCR. The most striking increase in transcripts was in VEGFR-3 and CD31 at 96 hr (Figure 5B). Coculture of HMS-1 with other breast carcinoma cell lines induced to grow as spheroids produced no appreciable increase in the transcripts (data not shown).

### Observational studies

**Figure 6.**
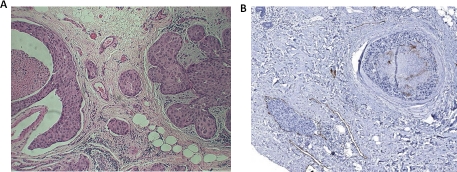
Histopathology of human breast carcinoma cases (A) Classic DCIS (right) juxtaposed to lymphovascular invasion (left) with no intervening stromal invasion. (B) Classic DCIS (upper right) with no intervening stromal invasion juxtaposed to a tumor embolus within a lymphatic space (lower left), the latter identified by D2-40 immunoreactivity.

The relevant clinicopathological information of the selected cases and age- and size-matched controls is depicted (Table 1). The 10 cases chosen for morphometric and immunocytochemical studies all showed prominent *in situ* carcinoma adjacent to florid LVI (Figure 6A; Figure 6B) (Table 1).

**Table 1.** Clinicopathologic features of the human cases exhibiting direct in situ LVI without stromal invasion compared to agematched and size-matched controls.

Feature	Cases	Controls	Significance
Breast appearance	----	----	----
Redness	0/10	0/10	p=.5
Edema	0/10	0/10	p=.5
Nipple retraction	0/10	0/10	p=.5
Mammographic appearance	----	----	----
Abnormal microcalcifications	9/10	8/10	p=.5
Abnormal density	9/10	9/10	p=.5
Size (diameter)	1-5 cm	1-5 cm	p=.5
Previous FNA	1/10	2/10	p=.1
Previous core biopsy	3/10	4/10	p=.1
Concurrent sentinel node procedure	4/10	6/10	p=.1
Subsequent sentinel node procedure	6/10	4/10	p=.1
Grade of DCIS	----	----	----
Low	1/10	2/10	p=.5
Intermediate	3/10	3/10	p=.5
High	6/10	5/10	p=.5
Stromal invasion	0/10	0/10	p=.5
Lymphovascular invasion	10/10	0/10	p=.01
Paget's disease of nipple	2/10	1/10	p=.5
Axillary lymph node status	----	----	----
Positive sentinel node	7/10	1/10	p=.01
Positive additional nodes	3/10	0/10	p=.01
Biomarker profile	----	----	----
ER +, PR +, Her-2/neu −	3/10	3/10	p=.5
ER +, PR −, Her-2/neu −	1/10	2/10	p=.5
ER −, PR +, Her-2/neu −	1/10	0/10	p=.5
ER−, PR −, Her-2/neu +	3/10	3/10	p=.5
Triple negative	2/10	2/10	p=.5
Surgical procedure	----	----	----
Lumpectomy	8/10	9/10	p=.5
Mastectomy	2/10	1/10	p=.5

**Figure 7.**
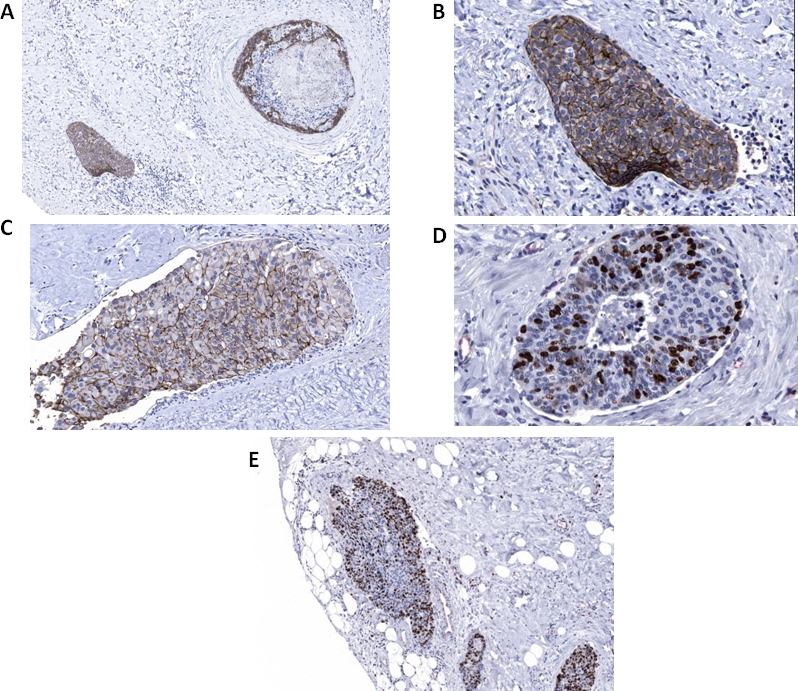
Immunocytochemistry of human breast carcinoma cases (A) Classic DCIS (upper right) and adjacent lymphovascular tumor embolus (lower left) exhibits E-cadherin immunoreactivity. (B) The lymphovascular tumor embolus in A (higher magnification) exhibits E-cadherin immunoreactivity. (C) DCIS transitioning to possible LVI exhibits E-cadherin immunoreactivity. (D) DCIS exhibits Ki-67 immunoreactivity. (E) The lymphovascular tumor embolus (left) exhibits Ki-67 immunoreactivity.

**Figure 8.**
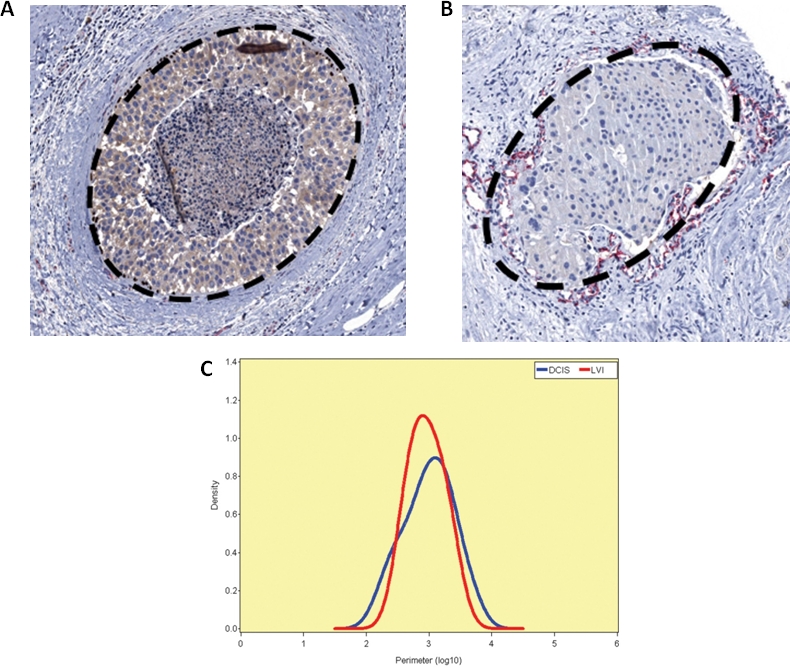
ImageJ algorithm (A) The ImageJ (National Institutes of Health, http://rsb.info.nih.gov/ij/) was used to “trace and measure” the perimeter of images of DCIS. 100 such profiles in 10 cases were captured. Other standard imaging algorithms were used to measure the elongation ratio and quantitate shape. (B) A similar number of lymphovascular tumor emboli were traced and measured. (C) The probability density function using a Gaussian kernel and the size distribution of the DCIS clusters versus tumor lymphatic emboli.

Exhaustive sectioning failed to reveal intervening stromal invasion. The immediate question which was raised by these observations was whether the *in situ* carcinoma cells had gained access to the lymphovascular space through an alternate non-invasive mechanism. The *in situ* clusters in all of these cases exhibited no statistically significant differences in E-cadherin membrane or Ki-67 nuclear proliferation immunoreactivities compared to those exhibited by the tumor emboli within the lymphovascular channels (p = 0.5; p = 0.5) (Figure 7A; Figure 7B; Figure 7C; Figure 7D; Figure 7E) (Table 2). The mean perimeter of the *in situ* clusters (1221 μ) did not differ significantly from that of the lymphovascular tumor emboli (1084 μ) (p=0.54) (Table 2) (Figure 8A; Figure 8B; Figure 8C). The *in situ* clusters differed only slightly in shape from the lymphovascular tumor emboli: the DCIS clusters tended to be round and smooth whereas the lymphatic tumor emboli were more oval and slightly irregular in shape. The elongation ratio of the *in situ* clusters was 0.85 ± .10 compared to the elongation ratio of the lymphatic tumor emboli which was 0.70 ± .2 but this difference was only minimally significant (p=0.05) (Table 2).

**Table 2.** Distribution of tumor marker immunoreactivity and morphometric values

Tumor Cluster^a^	DCIS	LVI	*t*-test
E-cadherin^b^	95±5%	93±6%	p=0.5
Ki-67^c^	23±4%	25±8%	p=0.5
Size (perimeter)^d^	1221μ±180	1084μ±160	p=0.1
Shape (elongation ratio)^e^	0.85±.10	0.70±.05	p=.05

a 100 clusters of DCIS and tumor emboli were individually analyzed for the given immunoreactivity or morphometric measurement and expressed as Mean ± SD. Differences were analyzed with the Student's *t*-test.

b E-cadherin immunoreactivity was measured as the percentage of tumor cells exhibiting membrane positivity.

c Ki-67 immunoreactivity was measured as the percentage of tumor cells exhibiting nuclear positivity.

d Size was measured as the perimeter of the DCIS clusters or lymphovascular tumor emboli and analyzed with ImageJ algorithms.

e Shape was measured as the elongation ratio (ratio of short to long axis) of the DCIS clusters or lymphovascular tumor emboli and analyzed with standard imaging algorithms.

These morphometric and immunocytochemical similarities exhibited by the *in situ* clusters and the lymphovascular tumor emboli suggested that these structures were one and the same and remained intact during their *in situ* to LVI transition. Only 4/10 cases had a prior FNA or core biopsy. No FNA or core biopsy tract was present in any of the areas of the *in situ* -LVI transition. Although all 10 cases eventually had a sentinel lymph node biopsy, only the 4 cases with a prior FNA or core biopsy had the sentinel lymph node procedure performed at the time of the excisional biopsy. The absence then of any antecedent physical manipulations in the majority of cases excluded mechanical disruption or an iatrogenic mechanism as the cause of this *in situ* -LVI transition. The clinicopathological characteristics exhibited by the cases showing this *in situ* -LVI transition were not dissimilar from the control *in situ* except for a disproportionately high degree of axillary nodal metastasis in the former (p=.01) (Table 1).

**Figure 9.**
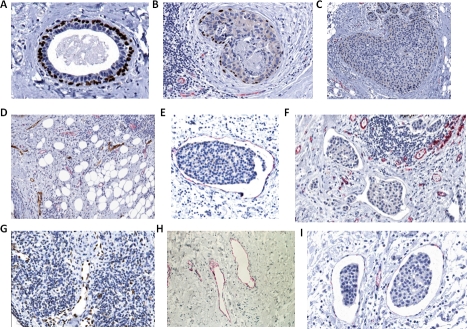
Additional immunocytochemistry of human breast carcinoma cases (A) Myoepithelial p63 immunoreactivity (brown) within normal ducts in the breast cancer sections. (B) Myoepithelial p63 immunoreactivity (brown) in DCIS and CD31 immunoreactivity (red) in adjacent lymphovascular spaces. (C) Myoepithelial p63 immunoreactivity (brown) in DCIS. (D) Double immunohistochemical immunoreactivity of D2-40 lymphatics (brown) and CD31 blood vessels (red). (E) CD31 immunoreactivity (red) in a lymphatic channel containing a tumor embolus. (F) CD31 and D2-40 negative immunoreactivities in two lymphovascular channels containing emboli and adjacent CD31 positive blood vessels (red). (G) Nuclear Prox-1 immunoreactivity (brown) within endothelial cells' lining of normal lymphatic channels. (H) Cytoplasmic/membrane VEGFR-3 immunoreactivity (red) within endothelial cells' lining of normal lymphatic channels. (I) VEGFR-3 immunoreactivity (red) in lymphatic channels containing tumor emboli.

In these cases, DCIS exhibited overall fewer numbers of surrounding p63 positive myoepithelial cells than normal ducts in the same sections (p=.01) (Figure 9A; Figure 9B; Figure 9C) (Table 3).

**Table 3.** Distribution of myoepithelial and endothelial immunoreactivity

Immunoreactivity^a^	0%	≤50%	>50%	*t*-test
p63 in normal ducts^b^	0	0	100%	
p63 in DCIS	4%	52%	44%	p=.01
CD31 in normal vessels	0	0	100%	
CD31 in LVI	75%	20%	5%	p=.01
D2-40^c^ in normal lymphatics	0	0	100%	
D2-40 in LVI	55%	34%	11%	p=.01
p63 in normal vessels/lymphatics	100%	0%	0%	
p63 in LVI	97%	Occasional cells	0%	p=.05
p63/CD31 in normal vessels	100%	0	0	
p63/CD31 in LVI	95%	Occasional cells	0%	p=.05
p63/D2-40 in normal lymphatics	100%	0	0	
p63/D2-40 in LVI	97%	Occasional cells	0%	p=.05

a A total of 100 ducts, lymphatics and blood vessels were separately counted. Immunoreactivity around these ducts and vessels is recorded as a percentage of circumferential immunoreactivity.

b Immunoreactivity for the designated marker(s) is recorded as percentage of 100 structures showing the indicated degree of circumferential immunoreactivity.

c The distribution of two other lymphatic endothelial markers, nuclear Prox-1 and cytoplasmic/membrane VEGFR-3 was similar to D2-40 (p=0.1).

In this analysis, we examined all types of vascular structures including lymphatics and blood vessels. Oftentimes it was difficult to distinguish lymphatics from blood vessels in routine sections. It was even difficult when using immunocytochemical methods as most single antibodies are not that specific in distinguishing blood vessels from lymphatics. In our hands we found that CD 31 was present in both blood vessels as well as lymphatics but that VEGFR-3, Prox-1 and podoplanin (D2-40) immunoreactivites were more specific for lymphatics. We used this combination of antibodies to support the conclusions of the study. Positive circumferential CD31 and D2-40 immunoreactivities meant immunoreactivities present along the lining circumference of the vessel which was further semiquantitated as being less ≤ 50% or >50% (Table 3).

Four adjacent lymphovascular populations were in evidence: blood vessels which were strongly CD31 positive and which were devoid of tumor emboli; lymphatics which were strongly D2-40, Prox-1 or VEGFR-3 positive and which were devoid of tumor emboli; lymphatics which were either less circumferentially D2-40, Prox-1 or VEGFR-3 positive or negative and which contained tumor emboli and lymphatics which were either less circumferentially CD31 positive or negative and which contained tumor emboli. There were differences in the the presence versus the absence and in the circumferential distribution of CD31 and D2-40, Prox-1 and VEGFR-3 immunoreactivities within the normal vessels in the breast cancer sections compared to those vessels containing tumor emboli (p=.01), (p=.01) (Figure 9D; Figure 9E; Figure 9F) (Table 3). To exclude subjectivity in perception or variations in immunocytochemical staining in different batch runs, we used the same magnification in the same field of view in the same section for many of the studies (Figure 9F).

**Figure 10.**
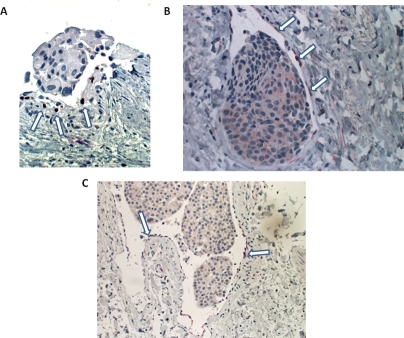
Double immunocytochemistry of human breast carcinoma cases (A) p63 nuclear immunoreactivity (brown) (arrows) in the lining cells of a tumor embolus-containing lymphatic channel. (B) Dual p63 nuclear (brown) (arrows) and CD31 membranous (red) immunoreactivities in a tumor embolus-containing lymphatic channel. (C) Dual p63 nuclear (brown) (arrows) and D2-40 membrane (red) immunoreactivities in a tumor embolus-containing lymphatic channel.

The CD31 and D2-40 immunoreactivities were more negative and less circumferential in the vessels containing tumor emboli. The same pattern of differences in the other lymphatic markers, eg., Prox-1 and VEGFR-3 in the normal lymphatics (Figure 9G, Figure 9H) versus the lymphatics containing tumor emboli (Figure 9I) was observed with both Prox-1 and VEGFR-3 similarly more negative and less circumferential in the lymphatics containing tumor emboli (p=0.01) (p=0.01). Whereas single p63, dual p63/CD31 and dual p63/D2-40 immunoreactivities were completely absent in normal vessels, in occasional CD31 immunostained, D2-40 immunostained or non-immunoreactive lymphatic channels containing tumor emboli, either single p63 myoepithelial immunoreactivity or dual myoepithelial and endothelial immunoreactivities were present (p=.05) (Figure 10A; Figure 10B; Figure 10C) (Table 3). Furthermore the dual immunoreactivities of p63/CD31 and p63/D2-40 were present within the same cells (Figure 10B; Figure 10C). In studies using single CD31 and D2-40 immunoreactivities, it was clear that CD31 recognized both blood vessels as well as lymphatics. However in the double immunocytochemical experiments, by using D2-40 as the first antibody and colorimetrically developing its target to brown before applying the second primary antibody, CD31, more effective discrimination between blood vessels and lymphatics could be achieved. Using both single as well as double immunostaining methods with CD31/D2-40, it was clear that the lymphovascular tumor emboli which were observed juxtaposed to DCIS were mainly within lymphatic channels. This was confirmed in both the Prox-1 as well as the VEGFR-3 immunocytochemical studies.

There was not much heterogeneity in the intensities of immunoreactivity exhibited by the cells positive for p63, CD31, D2-40, Prox-1 and VEGFR-3. The differences in these markers occurred with respect to their presence or absence and their degree of circumferential immunoreactivity. Some vascular structures and ducts showed no circumferential immunoreactivity, some showed partial (≤50%), which meant that less than or equal to one half of the circumference of the duct or vessel exhibited staining and some showed greater than 50% circumferential staining. The best way to record these patterns was semiquantitative. 100 sections of each structure were analyzed and results expressed as the percentage exhibiting 0% immunoreactivity (no staining), less than or equal to 50% circumferential staining or greater than 50% circumferential immunostaining. We did not count positive cells per se nor the intensity of staining. “Normal” refers to normal ducts or vessels present within the breast carcinoma tissue sections (Table 3).

The single p63 or dual p63/D2-40 and p63/CD31 immunoreactivities in occasional lymphatic channels containing tumor emboli and the more negative immunoreactivities of D2-40/CD31/Prox-1/VEGFR-3 in the majority of vessels containing tumor emboli suggested the possibility that they represented immature and newly created vasculature derived from myoepithelial-lined ducts. In these dual labeling studies, we considered using fluorescently tagged secondary antibodies instead of HRP-conjugated antibodies. However it would be technically difficult to carry out these latter experiments because of the high amount of autofluorescence present within paraffin-embedded archival material. If we had the opportunity to work with fresh or fresh frozen tissues, a fluorescent approach would, in fact, be both feasible and desirable.

**Figure 11.**
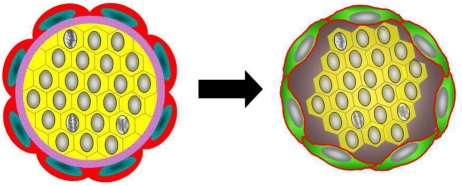
Schematic of encircling lymphovasculogenesis Schematic depicts encircling lymphovasculogenesis that results in tumor emboli formation.

Collectively the experimental as well as the observational studies suggested to us that the tumor emboli may have been the result of encircling lymphovasculogenesis rather than conventional lymphovascular invasion (Figure 11).

## DISCUSSION

Emerging experimental and clinical evidence has suggested that metastasis may be an earlier event in cancer progression than previously realized. Experimental studies in transgenic mice have observed the presence of breast cancer cells in the bone marrow even at the stage of atypical ductal hyperplasia or DCIS within the breast [1]. In observational studies in humans, both circulating tumor cells (CTCs) and disseminated tumor cells (DTCs) in the axillary lymph nodes and bone marrow have been noted in early stage disease, even at the stage of *in situ* carcinoma [28,29].

Observations made in experimental model systems should be validated in actual human tumors, if possible, in order to strengthen the relevance of the findings to humans. Observations such as epithelial-mesenchymal transition (EMT), autophagy, emergence of the multidrug resistance phenotype and vasculogenic mimicry are examples of important phenomena of tumor progression observed experimentally but only with modest confirmatory support in human cancers [15]. The findings of early metastasis in the experimental studies have begun to see some validations in the human situation. However more validations are certainly required. In the present study our experimental findings suggest cancer clusters can stimulate human myoepithelial cells or murine embryonal fibroblasts to engage in encircling lymphovasculogenesis which allows the clusters to become lymphovascular emboli. Our observational findings suggest that, in fact, *in situ* carcinomas can directly become lymphovascular tumor emboli. Both findings support the conclusion that early metastasis can occur in both an experimental animal model system as well as in humans.

Our hypothesis is that mammary *myoepithelial* cells or myoepithelial stem cells committed to the myoepithelial or fibroblast lineage transform to either endothelial cells or a commitment to the endothelial lineage. Our hypothesis to explain the genesis of tumoral emboli within lymphovascular channels is to consider the possibility that myoepithelial cells which surround clusters of DCIS, or stromal stem cells which lie adjacent to clusters of invasive carcinoma might, under the right circumstances, transform into encircling endothelial cells, causing the clumps of *in situ* or invasive carcinoma cells to become tumoral emboli.

In the experimental studies, the gross pattern of fluorescence observed in the collective experiments supported the conclusions that the unlabeled tumoral spheroids initially stimulated the growth of the labeled HMS-1 and MEFs which subsequently stimulated the influx of host cells within the tumoral extracellular matrix. On IHC examination the presence of GFP/RFP immunoreactivity within the cells which lined these lymphovascular channels containing the tumoral emboli supported our conclusions that the tumor cell clusters were inducing encircling lymphovasculogenesis from the injected HMS-1 or MEFs. This encircling lymphovasculogenesis eventually formed communicating anastomoses with murine vessels. This is why hybrid fluorescence emerged in the GFP-transgenics injected with unlabeled tumor cells and RFP-labeled HMS-1 cells or MEFs. We did not observe fluorescence or encircling GFP/RFP immunoreactivity when the HDFs were coinjected with the unlabelled spheroids. We interpret these findings to indicate that myoepithelial to endothelial or embryonal fibroblast to endothelial transformation may be mediated by stem/progenitor cells present in either the HMS-1 or the MEFs populations capable of pluripotency and the ability to switch to endothelial differentiation. It would not be unanticipated that an embryonal line like MEFs or a benign myoepithelial tumor cell line like HMS-1 would contain such pluripotent stem/progenitor cells. On the other hand, mature adult fibroblasts such as HDFs might lack such pluripotent cells and be incapable of encircling lymphovasculogenesis or a switch in differentiation.

In the mouse studies, the coinjected RFP-labeled HMS-1 or MEFs will always emit a red color and hence in the setting of the GFP-transgenics, the presence of anatomizing vessels is suggested by a yellow hybrid color. These experiments alone do not necessarily mean that the myoepithelial cells and embryonal fibroblasts developed into cells which lined the lymphovascular channels but taken together with the *in vitro* studies (Figure 4) and the other set of mouse studies showing GFP immunoreactivity in the cells lining the spaces (Figure 3), the aggregate studies suggested this possibility. The yellow hybrid color observed in the murine studies would not further transition into a green color because there would always be a combination of RFP-labeled HMS-1 or RFP-labeled MEFs and murine GFP-labeled endothelial cells or endothelial precursors in the tumor microenvironment giving the hybrid yellow fluorescence.

Even though our experimental model utilized spheroids or clumps of invasive breast cancer cells and not true DCIS, when coinjected or cocultured with human myoepithelial cells, the myoepithelial cells encircled the spheroids both *in vitro* as well as in mice in the manner in which myoepithelial cells lie juxtaposed to DCIS *in vivo*. When the spheroids were coinjected or cocultured with murine embryonal fibroblasts, these latter cells similarly encircled the spheroids both *in vitro* as well as in mice in the manner in which stromal fibroblasts lie juxtaposed to invasive ductal carcinoma. Both the human myoepithelial cells and the murine embryonal fibroblasts developed into cells which lined the lymphovascular channels containing the tumor emboli. In both situations then, the carcinoma cells induced an encircling lymphovasculogenesis.

It would be interesting if we could directly show that the presumed new lymphovascular structures transported fluid or cells. However, direct evidence for the above would require extensive imaging studies beyond the scope of the present study. However we believe that we have indirect evidence to support the above conclusions. The new lymphovascular structures labeled with GFP contained luminal erythrocytes indicating that they were transporting circulating red blood cells (Figure 3B). Furthermore murine studies with coinjected MARY-X spheroids and labeled HMS-1 or MEFs exhibited spontaneous pulmonary metastasis which could only occur if the newly created lymphovascular structures were able to communicate with the resident lymphovasculature and transport both fluid as well as tumor cells.

We are not claiming in this study that the MARY-X model was a model of DCIS. Breast carcinomatous emboli are indeed invasive carcinoma and it is our hypothesis that they may gain access to lymphovascular channels by stimulating the growth of the latter around them. To show this, we used a model of invasive carcinoma, MARY-X, that possessed the phenotype of florid lymphovascular invasion. In terms of the experimental model, the induction of lymphovasculogenesis takes origin from either murine embryonal stem cells (MEFs) or human myoepithelial cells (HMS-1) within the tumor microenvironment. In the observational studies we used cases in which DCIS was seen juxtaposed to areas of lymphovascular invasion without intervening stromal invasion. In both the murine experimental studies as well as the human observational studies, the common phenomenon of the induction of encircling lymphovasculogenesis is illustrated. But in the experimental studies it is invasive carcinoma doing the induction. In the observational studies it is the DCIS.

In the observational studies, we noted that *in situ* carcinoma lay juxtaposed to LVI without intervening stromal invasion in 10 cases. Even after an exhaustive search for stromal invasion, we could not demonstrate it. Four possible explanations might still be considered to support stromal invasion as being the mechanism of lymphovascular invasion in these 10 cases despite our not being able to demonstrate it: a) stromal invasion might have occured a long time before the tissue was sectioned; b) evidence for stromal invasion might be present in an adjacent piece of tissue that was not subjected to sectioning; c) a very small number of tissues was analyzed in the present studies; d) one can not draw positive conclusions based on negative evidence. However in response to these considerations: a) the vast majority of human breast cancers show obvious and florid stromal invasion with lymphovascular invasion being rare. In these cases, the stromal invasion remains and does not disappear as the tumor grows. The proposed reason of why the stromal invasion is absent in our 10 cases was that it once was there and has now disappeared. This possibility is just not tenable; b) exhaustive sectioning was done and certainly much more than the routine degree of sectioning which is done in pathological analysis. If you applied this possible consideration to all of diagnostic pathology, you could argue that disease processes, like cancer, are routinely missed because their presence falls outside the area of sectioning. Although this can rarely occur, it certainly does not commonly occur or otherwise diagnostic pathology would be completely unreliable. In the 10 cases of the present study, a deliberate and intense search for areas of stromal invasion was carried out. We sectioned all areas of the cancer (outside, midzone and inside) and failed to find stromal invasion; c) we have analyzed 10 cases to date in which we have observed this phenomenon. Although this may be perceived as a small number, we believe it to be a significant number because it is these exceptions that can prove the rule. In other words in the typical case of infiltrating breast cancer, the phenomenon of encircling lymphovasculogenesis may also be occuring but it is not possible to prove because of the presence of stromal invasion juxtaposed to lymphovascular invasion. In these typical cases, one has to presume that stromal invasion progresses to lymphovascular invasion. However in the 10 cases where there is the absence of stromal invasion but the presence of lymphovascular invasion, this presumption can be excluded and one is left with support for our hypothesis: encircling lymphovasculogenesis; d) Although it is true that absence of proof is not absolute proof of absence, we are not drawing positive conclusions solely on the basis of negative evidence. We have positive evidence: the presence of clusters of DCIS juxtaposed to clusters of tumoral emboli within lymphovascular spaces. Both clusters show similar size, proliferation and immunocytochemical features. That is circumstantial but positive evidence. It is the totality of evidence, both positive as well as negative, that supports our conclusions.

Furthermore lymphatic markers, eg., D2-40, VEGFR-3, Prox-1 were completely absent in normal ducts as well as ducts containing DCIS. Therefore we believe that we have excluded the possibility that lymphatic markers are being expressed by a subset of myoepithelial cells that represent ducts containing DCIS rather than lymphatics containing tumor emboli.

There was also no evidence for iatrogenic seeding. We reasoned that the only tenable hypothesis that would support these findings was one of induced encircling lymphovasculogenesis. In all 10 cases (Table 1), the morphometric and immunohistochemical evidence supported our hypothesis. It is important to note that the vast majority of DCIS in patients does not manifest LVI in the absence of stromal invasion. Most DCIS, when associated with progression, exhibits frank stromal invasion. LVI occurs after there is significant stromal invasion. But the findings of our present study indicate that this usual type of progression need not be canonical---that *in situ* carcinomas may progress to lymphovascular tumor emboli through an alternate “noninvasive” mechanism.

Because it could also be argued that the apparent tumoral emboli within lymphovascular spaces might, alternatively, be DCIS with separation artifact and a reduced or lost myoepithelial layer, we had to also consider this possibility. We do not believe that we are observing a retraction artifact phenomenon here. First of all, retraction artifact is usually observed in islands of invasive ductal carcinoma and not DCIS. Secondly, the DCIS clusters which were obvious were surrounded by p63 positive myoepithelial cells with no obvious separation artifact. The lymphovascular tumoral emboli were surrounded by CD31 and D2-40 positive endothelial cells. Thirdly the 10 cases of DCIS with LVI had metastases to axillary lymph nodes (Table 1). These findings all suggested that the LVI is true and not retraction artifact around DCIS, the latter of which would not give rise to axillary metastasis.

Our morphometric and tumoral IHC studies (Table 2) indicated no differences in immunoreactivity or size (perimeter) between the *in situ* clusters and the tumor emboli (p=0.5) (p=0.1). This suggested that these structures were one and the same and remained intact during their *in situ* to LVI transition.

With respect to this transition, the near identity of proliferation (Ki-67) and immunocytochemical adhesion markers (E-cadherin) further supported the hypothesis that these two structures were, in fact, related. If the structures were not related, that is if the DCIS occurred independently and the lymphovascular emboli were derived from invasive cells, it would be more likely that these markers within the emboli would change. Invasion is thought to involve epithelial-mesenchymal transition where there is loss of E-cadherin. Furthermore there is generally thought in tumor progression that there is clonal selection for a more aggressive phenotype with increased proliferation. It would then be expected that if the lymphovascular emboli were derived from stromal invasion, that their E-cadherin would be decreased and their Ki-67 would be increased compared to the DCIS foci. On the other hand, if the DCIS clusters and lymphovascular emboli were one and the same, it would be expected that E-cadherin and Ki-67 would be nearly identical between the two structures and that is exactly what was observed. Slightly different morphometric differences in shape between these structures, however, would not negate their identity of origin because the lymphovascular tumor emboli would likely be subjected to hydrostatic pressures of lymph and blood flow that could easily alter their shape. Our collective histological and immunocytochemical findings, though not proving our hypothesis, support it. Although there are alternative explanations, the one that is most consistent with Occam's razor is *in situ* – LVI transition.

Our hypothesis to explain the genesis of tumoral emboli within lymphovascular channels then is to consider the possibility that myoepithelial cells which surround clusters of DCIS or stromal cells which lie adjacent to clusters of invasive carcinoma might, under the right circumstances, transform into encircling endothelial cells, causing the clumps of carcinoma cells to become tumoral emboli. If one invoked the canonical hypothesis of invasion into pre-existing lymphatics and blood vessels to explain LVI, one would expect to see similarities in immunoreactivity between vessels containing and devoid of emboli since all of these vessels would have antedated the LVI. But our study observed CD31, D2-40, VEGFR-3 and Prox-1 differences in the embolic channels. Newly derived vascular channels would be expected to be immature and express endothelial markers, podoplanin (D2-40), VEGFR-3 and PECAM-1 (CD31), to a lesser degree than mature preexisting vessels (Table 3). If one invoked the invasion hypothesis to explain LVI, p63 immunoreactivity would be decreased in the DCIS-surrounding myoepithelial layer but not persistent within the CD31 and D2-40 lymphovascular spaces containing emboli. Our myoepithelial and endothelial IHC studies then also supported the concept of encircling lymphovasculogenesis.

If we limited our studies to the use of a single endothelial marker, we would have had to be more tentative in our conclusions. The use of single markers would limit the significance of our conclusions because in the tumor microenvironment slight changes might occur in the expression of a single gene, without implying any significance. While we did not exhaustively examine the expression of every known endothelial marker, in our experimental studies we studied the induction of VEGFR-1, VEGFR-2, VEGFR-3, CD31 and podoplanin, five different endothelial markers. In our human studies we studied the endothelial immunoreactivites for four markers, CD31, D2-40, VEGFR-3 and Prox-1. Random co-expression of several endothelial markers is unlikely and for this reason, we believe that our data supports our hypothesis. Nevertheless our findings could have been strengthened by the use of additional markers of lymphatic endothelium including neuropilin-2, FOXC2, CCR7, CCL19, CCL21 and the mannose receptor.

With these findings and this reasoning as background, we also observed lymphovasular channels with dual immunoreactivity. These channels showed CD31 and D2-40 immunoreactivities (red) as well focal p63 nuclear immunoreactivity (brown) (Figure 10). The focal brown nuclear immunoreactivity can be contrasted with adjacent non-immunoreactive blue endothelial and tumoral nuclei present within the same section. The use of confocal microscopy with immunofluorescent staining would be theoretically helpful in confirming these findings, but because this was a retrospective study using paraffin-embedded archival pathological materials, the degree of autofluorescence would confound meaningful interpretation of confocal immunofluorescent studies. We therefore, were not able to use this additional approach.

It had been known that myoepithelial cells exert tumor suppressive effects on DCIS [30-32]. More recently it had been demonstrated that myoepithelial cells can be paracrinely regulated by DCIS [33-38] and undergo alterations in gene expression, and promoter methylation [35,36]. In these studies the findings suggested that myoepithelial cells can become less differentiated and manifest aberrant gene expression including expression of endothelial-related genes [2,7,36,39,40,41]. In our *in vitro* experimental studies of the induction of endothelial differentiation within the myoepithelial cells, we observed an increase in podoplanin (the antigen recognized by D2-40), VEGFR-3, VEGFR-1, VEGFR-2 and CD31 transcripts over time when myoepithelial cells were cocultured with MARY-X spheroids. These studies suggest that endothelial (both lymphatic as well as vascular) proteins increase as the commitment to the endothelial lineage is made. Interestingly, we tested MARY-X for all the VEGF growth factors by real time PCR and it expressed relatively higher levels of VEGF-C and VEGF-D than VEGF-A and VEGF-B compared to a number of common ER positive and ER negative breast cancer cell lines. MARY-X would be expected therefore to stimulate lymphangiogenesis. Still the other breast carcinoma lines were not able to increase VEGFR-3, CD31 or podoplanin transcripts in HMS-1 cells, yet, in at least some of these other cell lines, VEGF-C transcripts were increased and presumbably VEGF-C was produced. The mechanism of induction of endothelial differentiation in myoepithelial cells may not therefore be mediated by the classic effectors of lymphangiogenesis, eg., VEGF-C.

We believe that we have provided supportive evidence for the acquisition of the lymphatic phenotype by myoepithelial cells *in vitro*. In the studies depicted in Figure 5, we examined the levels by RT-PCR of a number of endothelial markers in HMS-1 cells cocultured in media with MARY-X spheroids. At time 0, the expression of five different endothelial markers: VEGFR-1, VEGFR-2, VEGFR-3, CD31 and podoplanin was very low but each increased to various degrees when cocultured with MARY-X spheroids. The lymphatic endothelial markers, VEGFR-3 and podoplanin and the general endothelial marker, CD31 increased the most. We examined the presence of endothelial growth factors in MARY-X and compared the levels to other breast carcinoma lines and found that MARY-X makes high levels of VEGF-C. We did not measure the levels of VEGFR in MARY-X because this was not central to the present hypothesis and because in our analysis of the HMS-1 cells, there was no possibility of contamination by MARY-X. HMS-1 cells grew as a monolayer and the MARY-X spheroids grew in suspension without the ability to attach and the two cell lines are easily separated from each other. Furthermore at time 0 (Figure 5B), levels of VEGFR and other endothelial markers were low in HMS-1 cells and increased over time. This increase could only occur if there was an induction of endothelial differentiation of HMS-1 cells by the MARY-X spheroids. The rationale for studying the expression of VEGF in MARY-X is that we wanted to know what is different in MARY-X that might be responsible for its unique ability to induce encircling lymphovasculogenesis and possible candidates would include VEGF family members. VEGF-C, despite some conflicting data, represents an attractive candidate for future studies.

In the experimental models where induced vasculogenesis and/or vasculogenic mimicry has been observed [15-21], the phenomenon facilitates metastasis from the standpoint that the vascular channels which are created anastomose with the resident vasculature. We can not tell in our observational studies whether anastomoses are occuring as we can not see three dimensions in two dimensional sections.

Though the lymphovascular tumor emboli were of similar size as the *in situ* carcinoma clusters (Table 2), the lymphovascular channels containing the tumor emboli exhibited evidence of immaturity (Table 3). Channels derived from encircling lymphovasculogenesis would be expected to be immature. We can not directly validate or investigate this assumption, however, in our observational studies. However in our experimental *in vitro* induction studies of endothelial differentiation of myoepithelial cells, we observed an increase in podoplanin, VEGFR-3, VEGFR-1, VEGFR-2 and CD31 transcripts over time. These studies suggest that endothelial (both lymphatic as well as vascular) proteins increase as the commitment to the endothelial lineage is made during encircling lymphovasculogenesis.

In a recent study which observed strong correlation of extensive retraction artifact in the primary carcinomas with nodal metastases, it was postulated that the retraction artifact seen around tumor nests might be an early stage of LVI, where the conversion of mesenchymal cells to endothelial cells had not yet occurred [42]. Retraction artifact may also then be a reflection of early encircling lymphovasculogenesis.

Finally, it is known that “pure” DCIS can be associated with nodal metastases and explanations for this phenomenon have been proposed ranging from sampling bias, microinvasion misinterpretation, iatrogenic dissemination and “revertant DCIS”, a phenomenon where DCIS reverted back from invasive carcinoma [43]. In light of the data presented in the present study one might propose yet another explanation --- encircling lymphovasculogenesis to explain how initially “pure” DCIS might metastasize without invading.

In both our experimental as well as observational studies, we have not shown that myoepithelial cells or embryonal fibroblasts are capable of direct transdifferentiation into endothelial cells. Even though coinjection experiments with HMS-1 and MEFs in mice suggested that they can form lymphovascular channels via encircling lymphovasculogenesis and that immunocytochemical observations in human cases indicated p63 nuclear/CD31 membrane and p63/D2-40 dual immunoreactivities within the same lining cell, stem cells within HMS-1 or MEFs or within the breast undergoing a switch in differentiation from myoepithelial or fibroblast to endothelial could account for our findings.

We certainly do not know the mechanism for the induction of this encircling lymphovasculogenesis observed experimentally *in vitro* or in mice or in the *in situ*–LVI cases. The resemblance of the *in situ* carcinoma clusters, the lymphovascular tumor emboli and the spheroids to the embryonal blastocyst raises the possibility that embryonic lymphovasculogenic signaling pathways may be involved.

Our findings acknowledge the general importance of endothelial progenitor cells to tumor lymphovasculogenesis. Recent evidence, for example, has argued that some endothelial progenitor cells can reside in the bone marrow [20]. All endothelial cells do not need to take origin from pre-existing endothelial or endothelial precursor cells, however. And not every case of human cancer need involve active lymphangiogenesis [44,45]. But what we are arguing in this study is that active lymphovasculogenesis can occur and when it occurs, can occur from mesenchymal or myoepithelial precursors.

In these experimental studies we used MARY-X. Since MARY-X represents an unusual type of breast cancer with a somewhat unique gene expression profile, cellular properties and behavior *in vivo*, one might question the general applicability of this model to other common forms of breast cancer. If one focuses on the lymphovascular embolus, however, which is also present in non-IBC breast cancer, one might reason that the exaggerated phenotype of LVI exhibited by MARY-X provides a model to help understand the phenomenon of LVI exhibited by common forms of breast cancer.

Our use of the term “encircling lymphovasculogenesis” must be distinguished from “circumferential lymphangiogenesis”, which has been used to describe a different process [46]. This latter term refers to tumor-dependent induction of lymphangiogenesis in peritumoral normal tissue surrounding the tumor mass. Our definition of the former term is the development of lymphovascular channels which envelop or encircle tumoral clumps creating lymphovascular tumoral emboli. The mechanisms behind “encircling lymphovasculogenesis” and “circumferential lymphangiogenesis” may be similar, however.

The occurrence of encircling lymphovasculogenesis does not negate, however, the generally accepted phenomenon of classic lymphovascular invasion which may still represent the usual and dominant route of metastasis. However this pathway of classic lymphovascular invasion need not be obligate. Alternate pathways such as encircling lymphovasculogenesis may also be operating.

Collectively our experimental as well as our observational studies suggest that breast cancer tumor emboli may result from encircling lymphovasculogenesis rather than conventional lymphovascular invasion. This phenomenon may help short-circuit some of the steps of the metastatic process which may indeed give rise to “early metastasis”.

## MATERIALS AND METHODS

### Ethics Statement

This study was approved by The Ohio State University Cancer Institutional Review Board (IRB) under protocol 2006C0042. All animal studies and *in vitro* experimental studies were approved specifically by The Ohio State University's Animal Care and Use Committee (IACUC), protocol 2007A0218 and by The Ohio State University's Institutional Biosafety Committee, protocol 2007R0057. Use of human tissues was specifically approved by The Ohio State University Cancer Institutional Review Board (IRB) under protocol 2006C0042.

### Experimental studies

#### Cell lines

We used a previously established xenograft model of inflammatory human breast cancer (MARY-X), a model that exhibited a nodular growth pattern centrally and florid LVI peripherally and which generated tumoral spheroids *in vitro*. [22]. Inflammatory breast cancer is a unique type of breast cancer with a distinct genotypic and phenotypic profile [23-25]. The establishment of this xenograft model occurred in the 1990's when the investigator was at the University of California at Los Angeles (UCLA) and was approved by the UCLA's Institutional Review Board at that time. The human myoepithelial cells were obtained from a previously established human myoepithelial cell linec (HMS-1) derived from a benign salivary gland tumor [26] also established in the 1990's when the investigator was at UCLA and was also approved by UCLA's Institutional Review Board at that time. The murine embryonal fibroblasts (MEFs) were purchased (American Type Culture Collection (ATCC), Manassas, VA) and adult human dermal fibroblasts (HDFs) derived from human adult skin, gifted by Andrew C. Issekutz, Dalhousie University, Halifax, Canada. All other lines consisted of the estrogen receptor (ER) negative breast cancer lines (MDA-MB-231, MDA-MB-468), the ER positive line, MCF-7 and the Her-2/neu amplified breast cancer line, HTB20, all also purchased (American Type Culture Collection (ATCC), Manassas, VA). All the cell lines were grown under standard conditions in Dulbecco's Modified Eagle Medium (DMEM) with 10% fetal bovine serum (FBS) with the exception of the fibroblast lines which were grown in Minimal Essential Medium (MEM)-alpha with 10% FBS and the myoepithelial lines which were grown in keratinocyte serum free media (KSFM) with supplements (Life Technologies, Inc., Gaithersburg, MD).

#### Transfections

A retroviral expression vector pLNCX2-DsRed or pLNCX2-zsGreen containing enhanced green fluorescent protein (GFP) or red fluorescent protein (RFP) respectively was used to transfect a retroviral packaging cell line, RetroPack PT67 (Clontech Laboratories, Inc., Mountain View, CA). The viral supernatants were harvested 72 hours after transfection. The filtered undiluted retroviral supernatants were used immediately to infect the target cells, HMS-1 or MEFs which were seeded in 60-mm plates. After 48 hr, antibiotic G418 (500-1000 μg/ml) was added to select the clones. After 1-2 weeks, positive clones exhibiting intense fluorescence emerged and were monitored with the Fluorescence Imaging Microscopy-Nikon Eclipse TE2000-U System.

We labeled both HMS-1 and MEFs with both GFP and RFP and conducted different combinations of experiments with these labeled lines. We chose two tags and two cell lines because we wanted to see both a single pattern of fluorescence in nude mice (using GFP-labeled HMS-1 or MEFs or RFP-labeled HMS-1 or MEFs) or a double pattern of fluorescence in GFP-transgenic nudes (using RFP-labeled HMS-1 or MEFs). We chose two cell lines because we wanted to see whether either human myoepithelial cells, ie HMS-1 or murine embyonal fibroblasts, ie. MEFs could transform into endothelial cells when coinjected with MARY-X spheroids. We wanted to see whether encircling lymphovasculogenesis could be induced.

#### Antibodies and general reagents

Two different monoclonal antibodies to human and murine CD31 respectively included a rat monoclonal anti-human specific CD31 anti-body (PharMingen, San Diego, CA) and a rat monoclonal anti-murine specificCD31 (PharMingen). A rabbit polyclonal anti-vascular endothelial growth factor-3 (VEGFR-3) (ab14828) (Abcam, Inc., Cambridge, MA) was also obtained. Secondary antibodies included Texas Red conjugated goat anti-rat (Jackson ImmunoResearch Laboratory, West Grove, PA). and Texas red conjugated goat anti-rabbit (Sigma-Aldrich, St. Louis, MO). Primary and secondary antibodies were used according to the manufacturers' recommendations. Matrigel matrix was obtained and used according to the supplier's guidelines (BD Biosciences, Bedford, MA).

#### Coculture experiments

Unlabelled spheroids generated from MARY-X [22] were cocultured with either GFP-labeled HMS-1 or MEFs or unlabeled HMS-1 in monolayer culture or in Matrigel. Approximately equal volumes of each cellular population was added to this mixture. We used Matrigel in the *in vitro* experiments because we needed a three dimensional scaffold to hold the MARY-X spheroids in place which would allow the cocultured HMS-1 cells or MEFs to undergo chemotaxis or haplotaxis toward the spheroids, eventually encircling them. Without this Matrigel scaffold, this phenomenon could not occur as the monolayers of HMS-1 and MEFs would not be able to reach the spheroids which would remain in suspension culture. In the Matrigel cocultures involving HMS-1, anti-human specific CD31 antibody was added followed by Texas Red conjugated goat anti-rat. In the Matrigel cocultures involving MEFs, anti-murine specific CD31 antibody was added followed by Texas Red conjugated goat anti-rat. The Matrigel cocultures were observed over the next 24-96 hours by both phase contrast and fluorescence microscopy. For immunocytochemical staining, the cells of the Matrigel coculture were fixed in cold methanol: acetone (1:1) for 20 minutes. All the staining was carried out in blocking buffer. The stained cells were imaged on the Nikon Fluorescent microscope using the Roper camera and Metavue software (Universal Imaging Corporation, West Chester, PA).

In the monolayer coculture experiments with MARY-X spheroids in suspension and unlabeled HMS-1 cells as a monolayer, the spheroids which remained in suspension were separated from the monolayers of HMS-1 after 24, 48, 72 and 96 hours. RNA was extracted from each cellular population obtained at these time intervals.

#### RNA Isolation and cDNA Synthesis

The total RNA was isolated from cultured cells or tissues using RNeasy Mini Kit (Qiagen, Inc, Valencia, CA) per manufacturer's instructions. SuperScript III First-Strand Synthesis System (Invitrogen Corporation, Inc., Carlsbad, CA) was used for the first strand cDNA synthesis.

#### Real time PCR

An aliquot of 20 ng cDNA was used in each 25 μl PCR reaction, using Platinum Taq DNA Polymerase High fidelity (Invitrogen Corporation, Inc.). The following conditions were used: denaturation at 94°C for 30 seconds, annealing at 58°C for 30 seconds, and extension at 68°C for 1 min for a total of 25, 30 or 35 cycles. PCR products were analyzed by 2.0% agarose gel. Real-time PCR was performed on a ABI 7500 Real-Time PCR System (Applied Biosystems, Inc., Foster City, CA). cDNA was combined with primer sets and Power SYBR® Green PCR Master Mix (Applied Biosystems, Inc) was used. Gene expression levels were calculated relative to the housekeeping gene β-actin by using 7500 System SDS software (Applied Biosystems, Inc.).

Primer sets (forward and reverse) used for real time PCR included the following (forward, reverse), all to human transcripts:

VEGF-A, 5′-CTTGCCTTGCTGCTCTACC-3′, 5′-CACACAGGATGGCTTGAAG-3′

VEGF-B, 5′-AGCACCAAGTCCGGATG-3′, 5′-GTCTGGCTTCACAGCACTG-3′

VEGF-C, 5′-TGCCGATGCATGTCTAAACT-3′, 5′-TGAACAGGTCTCTTCATCCAGC-3′

VEGF-D, 5′-GTATGGACTCTCGCTCAGCAT-3′, 5′-AGGCTCTCTTCATTGCAACAG-3′

CD31, 5′-AGTGGTTATCATCGGAGTG-3′, 5′-TCATTTATTGGTTTCATT-3′

Podoplanin, 5′-GGAAGGTGTCAGCTCTGCTC-3′, 5′-CGCCTTCCAAACCTGTAGTC-3′

VEGFR-1, 5′-TTTTACCGAATGCCACCTC-3′, 5′-GCGTGCTAGCTGGATGTCTT-3′

VEGFR-2, 5′-GAACCAAATTATCTCCATCTT-3′, 5′-GCACTCCAATCTCTATCAGC-3′

VEGFR-3, 5′-AGCCATTCATCAACAAGCCT-3′, 5′-GGCAACAGCTGGATGTCATA-3′

ACTB, 5′-GGCACCCAGCACAATGAAG-3′, 5′-GCCGATCCACACGGAGTACT-3′

Real time PCR experiments were repeated 5 times and results of relative mRNA levels depicted as mean ± standard deviation.

#### Animal experiments

Approximately 1x10^7^ GFP-or RFP-labeled HMS-1, MEFs or HDFs were mixed with the MARY-X spheroids on an equal volume basis and injected subcutaneously into nude mice (*nu/nu* mutants on a BALB/c background) or GFP-transgenic nude mice (Anticancer, Inc., San Diego, CA.) in groups of 10. In these murine experiments and the previously mentioned *in vitro* coculture experiments, the conditions for mixing HMS-1 or MEFs with MARY-X spheroids were expressed in volumes rather than cell numbers. Because MARY-X forms spheroids which are very tight aggregates of tumor cells (approximately 10^3^ in 100μ diameter spheroid) and we did not disadhere the spheroids into individual cells in our coculture or coinjection experiments, we thought it more accurate to express relative amounts of cells added in volume rather than cell number terms. Furthermore the HMS-1 or MEFs are not in spheroids but exist singly from a monolayer and they are made into a pellet whose density is much less than that of the spheroids. In both the *in vitro* as well as the murine experiments, we mixed an equal volume of HMS-1 or MEFs with the MARY-X spheroids but because the MARY-X spheroids are at a much higher cell density there are approximately 20 fold more MARY-X cells than HMS-1 or MEFs in the same volume.

The injected mice were monitored for tumor growth by whole-body fluorescent imaging which was performed in a fluorescent light box (LT-9MACIMSYSPLUS system) illuminated by fiber-optic lighting (LT-9PANSEE system) equipped with two 470/40nm excitation filters for dual fluorescent light (green and red) (Lightools Research, Encinitas, CA). At selected intervals ranging up to 2 months, the mice were euthanized with 25 mg/ml ketamine, 2 mg/ml xylazine and acepromazine and subjected to necropsy. Ertirpated tumors were processed for routine histological examination as well as IHC with rabbit anti-GFP and anti-RFP polyclonal antibodies (BioVision Research Products, Inc., Mountain View, CA) followed by secondary antibody and DAB chromogenic detection.

### Observational studies

#### Selection and retrieval of cases

10 cases exhibiting DCIS with adjacent LVI but without stromal invasion were studied. The cases were exhaustively sectioned to exclude stromal invasion. As controls, age-matched and size-matched cases of DCIS, none of which exhibited LVI were selected for comparative studies.

#### Image Acquisition

The hematoxylin and eosin and immunocytochemical slides were scanned into virtual slides. Image acquisition utilized the Aperio ScanScope T2 scanner (Aperio, Vista, CA) producing images with a resolution of 20 pixels/10 μ.

#### Immunohistochemistry

The immunohistochemistry (IHC) studies utilized tumor proliferation (Ki-67) and adhesion (E-cadherin) markers singly; and myoepithelial (p63) and lymphovascular (podoplanin [D2-40], CD31, Prox-1, VEGFR-3) markers both singly and in combination, the latter combination employing double immunohistochemical staining [27].

The primary antibodies used were as follows: Ki-67 (clone MIB-1, Dako catalog number M7240), E-cadherin (clone NCH-38, Dako catalog number M3612), p63 (clone 4A4, NeoMarkers, catalog number MS-1081-P1), D2-40 (clone D2-40, Dako, catalog number M3619), VEGFR-3 (ab14828) (rabbit polyclonal, Abcam, Inc., Cambridge, MA), Prox-1 (clone 5G10, Millipore, Inc., Billerica, MA) and CD31 (rabbit polyclonal, catalog number E11114, Spring Bioscience, Fremont, CA).

For the single immunostaining methods (Ki-67, E-cadherin, D2-40, CD31, VEGFR-3, Prox-1), the primary antibody was diluted 1:100 and incubated for 30 minutes at room temperature. For Ki-67, E-cadherin and D2-40, the detection system used was a labeled Streptavidin-Biotin Complex. DAB chromogen was applied to develop the color. As controls for non-specific staining, we used negative isotype controls in all of our IHC experiments. For CD31 and VEGFR-3, the detection system, MACH 4 (Biocare Medical catalog M4U536L) was incubated for 30 minutes. Vulcan Fast Red was used to develop the color.

We carried out double staining for a number of different combinations of antibodies. These included p63/CD31, p63/D2-40, p63/VEGFR-3, D2-40/CD31. Prox-1 was used in only the single immunostaining and not the double immunostaining. The double staining procedure was always the same: The first antibody was used at the manufacturer's suggested dilution. The detection system, used was envision plus dual link for the first antibody (Dako code K4061). Lastly, DAB chromogen was applied to develop the signal of the first target. The second antibody was also used at the suggested manufacturer's dilution. The detection system, MACH 4, was used for the second antibody. Vulcan Fast Red was used to develop the signal of the second target.

For p63/D2-40 and p63/VEGFR-3 double staining, the p63 antibody was used first at a dilution of 1:500 and incubated for 30 minutes at room temperature. The detection system, envision plus dual link (Dako code K4061) was incubated for 30 minutes. Lastly, DAB chromogen was applied to develop the p63. D2-40 or VEGFR-3 was used at a dilution of 1:200 and incubated for 30 minutes at room temperature. The detection system, MACH 4, was incubated for 30 minutes. Vulcan Fast Red was used to develop either the D2-40 or the VEGFR-3 so that the two primary antibodies recognizing either myoepithelial or lymphovascular differentiation could be easily differentiated. For the D2-40/CD31 double staining, the D2-40 antibody was used as the first antibody. This was so because while D2-40 is thought to recognize lymphatics and not blood vessels, CD31 can recognize both lymphatics as well as blood vessels. By using D2-40 as the first antibody and colorimetrically developing its target to brown before applying the second primary antibody, CD31, more effective discrimination of lymphatics from blood vessels could be achieved.

#### Morphometric and Quantitative IHC Analysis

100 examples of each of the following were imaged and studied: ductal structures identified by circumferentially positive p63 nuclear myoepithelial immunoreactivity, DCIS clusters identified by morphological criteria, and at least partially circumferential p63 immunoreactivity; blood vessels and/or lymphatics identified by positive circumferential CD31 endothelial membrane immunoreactivity; lymphatics identified by positive circumferential D2-40 membrane immunoreactivity, Prox-1 nuclear immunoreactivity or VEGFR-3 cytoplasmic/membrane immunoreactivity; tumor emboli within vascular channels identified by morphology and/or at least partially circumferential CD31, D2-40, VEGFR-3 or Prox-1 immunoreactivity. The degrees of tumoral E-cadherin and Ki-67 immunoreactivities were quantitated from the virtual slides. Similarly the presence of single p63, CD31, D2-40, VEGFR-3 or Prox-1 or dual p63/D2-40, p63/CD31, p63/VEGFR-3 and D2-40/CD31 immunoreactivities within the lining myoepithelium or endothelium was quantitated. ImageJ [National Institutes of Health, http://rsb.info.nih.gov/ij/] was used to measure the perimeters of DCIS clusters and tumor emboli captured from scanned slides. Other standard imaging algorithms were used to measure the elongation ratio (ratio of short to long axis) of the DCIS clusters and the tumor emboli.

#### Statistics

Results of all *in vitro* and animal experiments and IHC measurements on the human tissue sections of the different groups (DCIS versus tumor emboli) were analyzed statistically with the two tailed Student's t-test as well as with an Analysis of Variance. Univariate kernel density estimation was performed for each perimeter measurement (ducts and vessels). The univariate kernel density is a measure of size distribution and is a standard way used to compare the distributions of two apparently different populations, in this case ducts containing DCIS and vessels containing tumoral emboli to investigate the similarity or dissimilarity of these two populations. The univariate kernel density was suggested to us by the Center for Biostatistics at Ohio State University.

## Abbreviations used

ATCC: American Type Culture Collection
CTCs: circulating tumor cells
DCIS: ductal carcinoma *in situ*
DMEM: Dulbecco's modified Eagle medium
DTCs: disseminated tumor cells
EMT: epithelial mesenchymal transition
ER: estrogen receptor
GFP: green fluorescent protein
HDFs: human dermal fibroblasts
HMS: human matrix secreting
IRB: institutional review board
LVI: lymphovascular invasion
MEFs: murine embryonal fibroblasts
MEM: minimal essential medium
PCR: polymerase chain reaction
RFP: red fluorescent protein
VEGFR-3: vascular endothelial growth factor receptor-3
